# Supplementary material for: A highly conserved tRNA modification contributes to C. albicans filamentation and virulence
Source: Microbiol Spectr. 2024 Apr 8;12(5):e04255-22. doi: 10.1128/spectrum.04255-22 (PMC11064501; doi:10.1128/spectrum.04255-22)
Supplement: Supplemental material — Supplementary Figures S1-S8 and supplementary Tables S1 and S2. [file spectrum.04255-22-s0001.docx]

**Table S1** Primers used in this study

| **Primer** | **Sequence (5`→3`)** |
| --- | --- |
| IF2115_1: | TACCGTCGAC**CTCGAG**TGTATAGATAGATGTACCGAAT |
| IF2115_2: | AAAAGCTGGGTACC**GGGCCC**CGTTGATGAAAAAGAAACAGAG |
| IF2114_1: | TACCGTCGAC**CTCGAG**ATAGTGTTAATATATGTTTGCGG |
| IF2114_2: | AAAAGCTGGGTACC**GGGCCC**CTAAGGATGATGGACCCAAA |
| IF2113_1: | TACCGTCGAC**CTCGAG**ACAAGAAAAAGTCACACGCAGG |
| IF2113_2: | AAAAGCTGGGTACC**GGGCCC**GTTGTTGATTGAGAGAAAACTAGA |
| CdUra14 | CTCCATTCCCAGTAACACCA |
| M13 rev | CAGGAAACAGCTATGACC |
| 2115_3 | GAACA**gGgcCc**TTCATTCAAGCACGAAGGGC |
| 2115_4 | TATAG**cTCgaG**GTGACATCTAAGGATGATGGAC |
| 2115_5 | GAAAAA**cCgcgg**TTTTATTTGCTCGCAACTCCC |
| 2115_6 | GAAAG**gAGctC**CCATGGTTGCATGCTCGTTC |
| 2115_7 | GCTTTTTCTATA**ggatcc**TTATCTAAACTGAG |
| 2115_8 | CAATTTTTTAT**gcGgcCGC**AACTC |
| 2115_9 | GTTGCAT**cCgCGg**TCTTTGTCTAC |
| 2115_8G | CTATACAC**ggaTcc**TCTAAACTGAG |
| 2115_9G | TTATTTGC**ctGCAg**CTCCCAA |
| 15150_3 | TCATTC**ggGCcC**GAAGGGCCAGAAACCAGACG |
| 15150_4 | CAGAT**ctcgag**ACAGATCTTGGTGACATTTGAAGA |
| 15150_5 | GTTTC**ccgcgg**AGGGTTTTGGCGTTGCATTC |
| 15150_6 | CATTT**gagcTc**ACCTGCCTTGACTCCAAACT |
| 15150_6c | CATTT**gagcTcAgCTG**CCTTGACTCCAAACT |
| 15150_7 | TGTCT**cccggg**TTATCTAAACTG |

Lowercase letters are nucleotide exchanges to create the bold restriction sites. Underlined letters show a second restriction site.

**Table S2** ct^6^A detection in *C. albicans* and *C. dubliniensis*

| *C. albicans* | Total Runs | ct^6^A Signal | Percent Positive |
| --- | --- | --- | --- |
| WT SC5314 YPD 30°C | 10 | 10 | 100% |
| Revertant YPD 30°C | 9 | 9 | 100% |
| WT SC5314 YPD 37°C | 9 | 9 | 100% |
| Revertant YPD 37°C | 9 | 9 | 100% |
| WT SC5314 Serum | 6 | 6 | 100% |
| Revertant Serum | 7 | 6 | 86% |
| **Total** | **50** | **49** | **98%** |
|  |  |  |  |
| *C. albicans deletion strains* | |  |  |
| 2115A YPD 30°C | 10 | 0 | 0% |
| 2115B YPD 30°C | 8 | 0 | 0% |
| 2115A YPD 37°C | 9 | 0 | 0% |
| 2115B YPD 37°C | 9 | 0 | 0% |
| 2115A Serum | 8 | 2 | 25% |
| 2115B Serum | 6 | 0 | 0% |
| **Total** | **50** | **2** | **4%** |
|  |  |  |  |
| *C. dubliniensis* |  |  |  |
| Wue284 YPD 30°C | 10 | 10 | 100% |
| Wue284 YPD 37°C | 9 | 9 | 100% |
| Wue284 Serum | 8 | 8 | 100% |
| **Total** | **27** | **27** | **100%** |


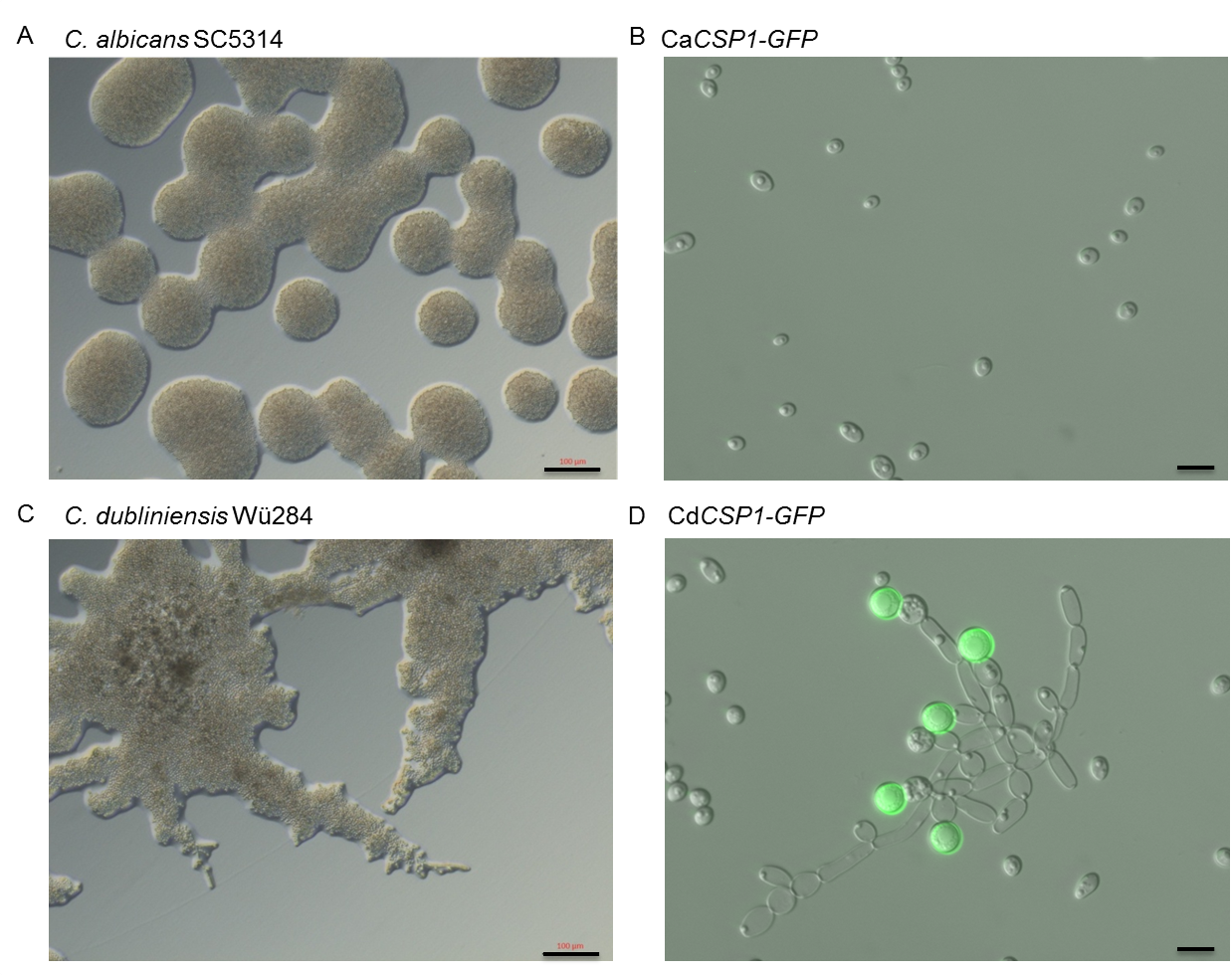


**Figure S1** Colony and single cell morphology of *C. albicans* and *C. dubliniensis* on SLAD-agar

*C. albicans* wild type SC5314 (A)*, C. dubliniensis* wild type Wü284 (C) and the chlamydospore-specific reporter strains *CaCSP1-GFP* (B) and *CdCSP1-GFP* (D) were grown in liquid SD-medium overnight and spread onto SLAD-agar. Agar plates were incubated three days at 30 °C in the darkness. Pseudohyphae and chlamydospore formation was exclusively observed by *C. dubliniensis* strains. *CdCSP1-GFP* produced frequently end-terminal fluorescent chlamydospores, whereas *C. albicans* remained in yeast form. Thus, no fluorescence signal was detectable using *CaCSP1-GFP* strain. The scale bar represents 100 µm in pictures A and C and 10 µm in pictures B and D.


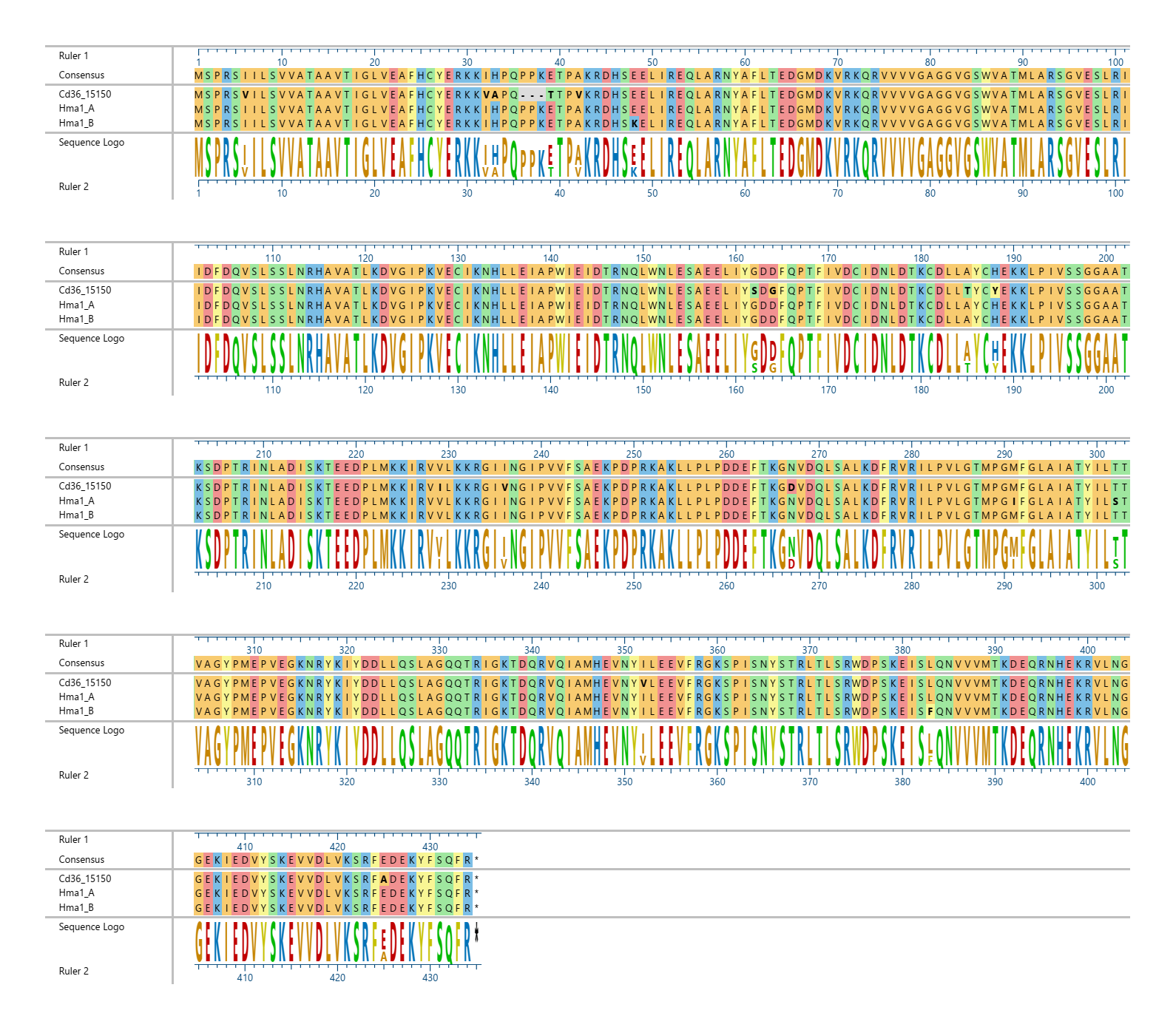


**Figure S2** ClustalW Alignment of *C. dubliniensis* Cd36_15150 protein with both *C. albicans* Hma1 alleles

Sequences as stored in the *Candida* genome database (www.candidagenome.org, downloaded July 2023) were aligned by ClustalW. Consensus sequence and sequence logo show the near identity between the sequences. Note that Hma1 has two alleles, A and B, in the phased *C. albicans* reference genome, with some minor non-synonymous sequence variants.


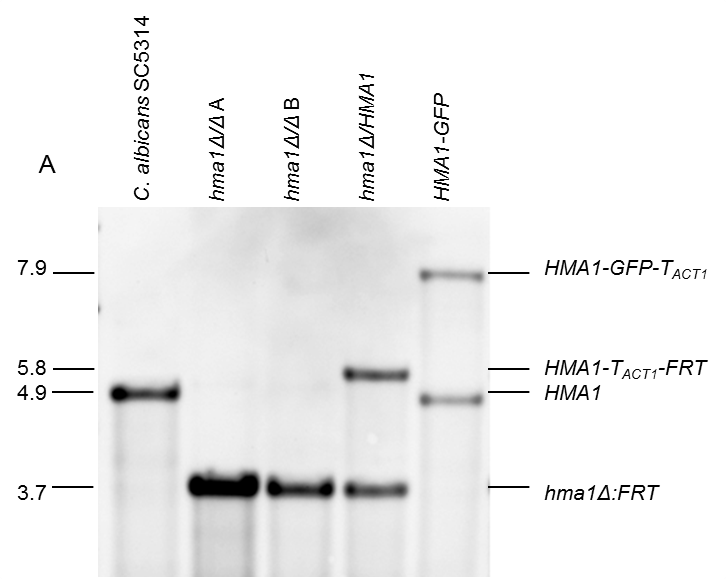

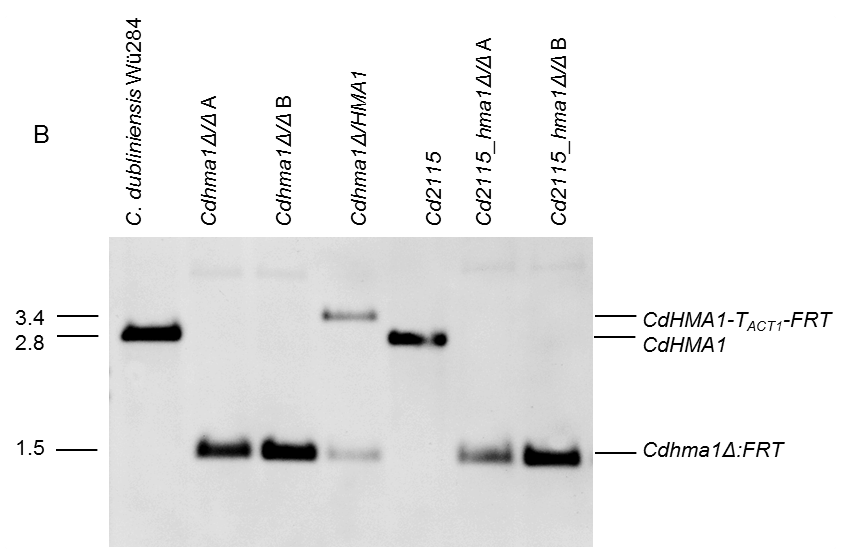


**Figure S3 Confirmation of strains**

(A) Confirmation of *C. albicans* strains. Southern-Hybridization: *EcoR*I cut gDNA of the wild type SC5314 (lane 1), the homozygous mutants *hma1*Δ/Δ A/B (lane 2/3), the complemented strain *hma1*Δ*/HMA1* (lane 4) and the *HMA1-GFP*-fusion strain (lane 5) with the *HMA1*-downstream specific probe. The band identities are named at the right border and the gDNA fragment sizes (in kb) are indicated at the left. (B) Confirmation of *C. dubliniensis* strains. Southern-Hybridization: *Sph*I cut gDNA of the wild type Wü284 (lane 1), the homozygous mutants Cd*hma1*Δ/Δ A/B (lane 2/3), the complemented strain Cd*hma1*Δ*/HMA1* (lane 4), the Cd*2115* strain (lane 5) and homozygous mutants Cd2115_*hma1*Δ/Δ A/B (lane 6/7), with the Cd*HMA1*-upstream specific probe. The band identities are named at the right border and the gDNA fragment sizes (in kb) are indicated at the left.


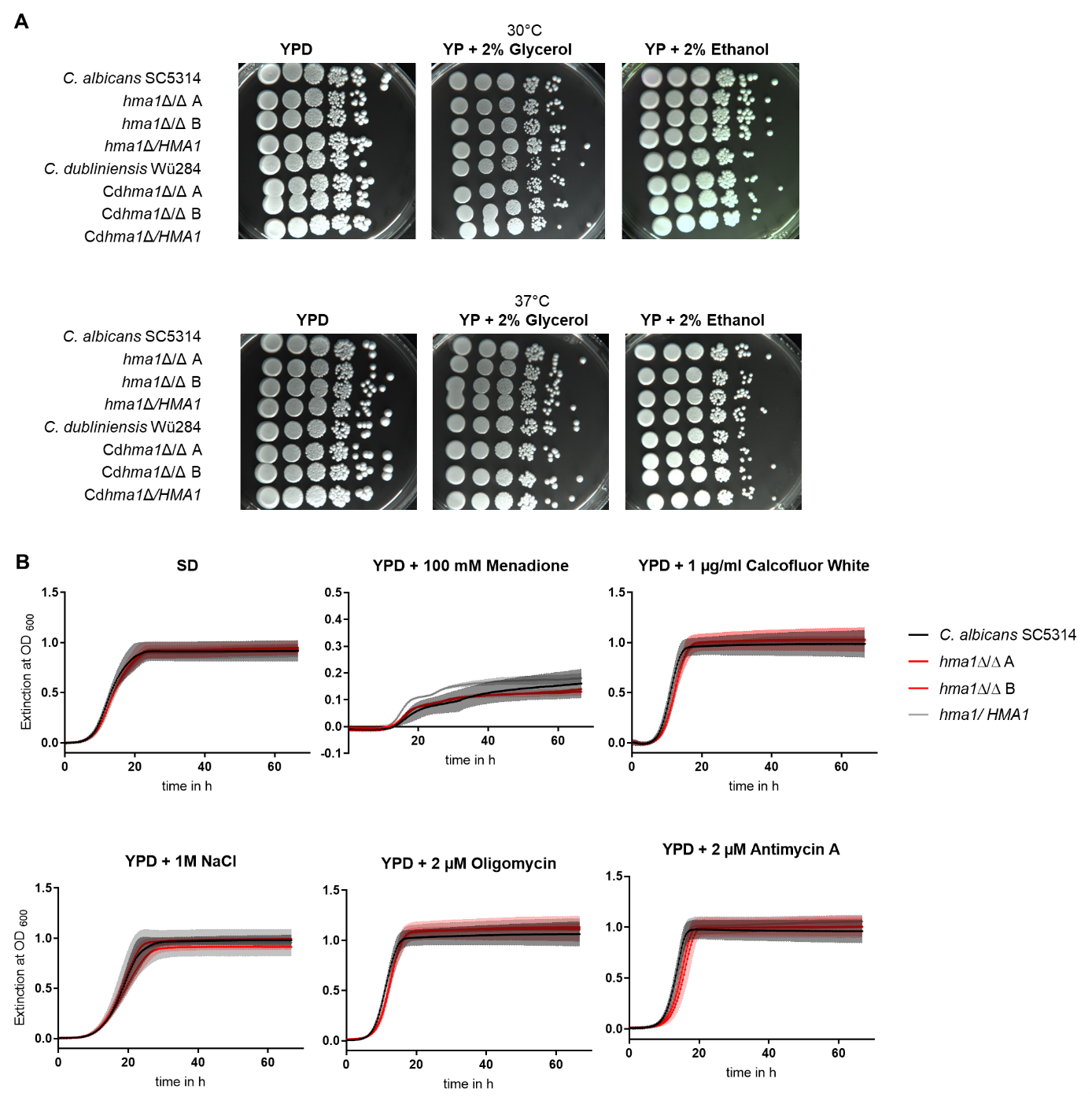


**Figure S4** Stress resistances test of *C. albicans hma1*Δ/Δ and *C. dubliniensis* Cd*hma1*Δ/Δ strains

1. 10^7^ to 10^2^ *Candida* cells were dropped onto YP-agar plates containing 2% glucose or the fermentable carbon sources 2% glycerol or 2% ethanol. Plates were incubated for 3 day at 30°C or 37°C.
2. *C. albicans* SC5314 and *hma1*Δ/Δ were grown overnight in YPD medium at 30°C. For preparation of growth curve assay cells were washed and set to an optical density of 0.01 in 200 µl medium in 96-well plates. Growth at 30°C of *C. albicans* strains was monitored up to 65 h in SD or YPD plus 100 mM menadione, 1 µg/ml calcofluor white, 1 M NaCl, 2 µM oligomycin or 2 µM antimycin A. Graphs show the mean ± SD of three independent biological replicates and two independently created *C. albicans* *hma1*Δ/Δ strains.


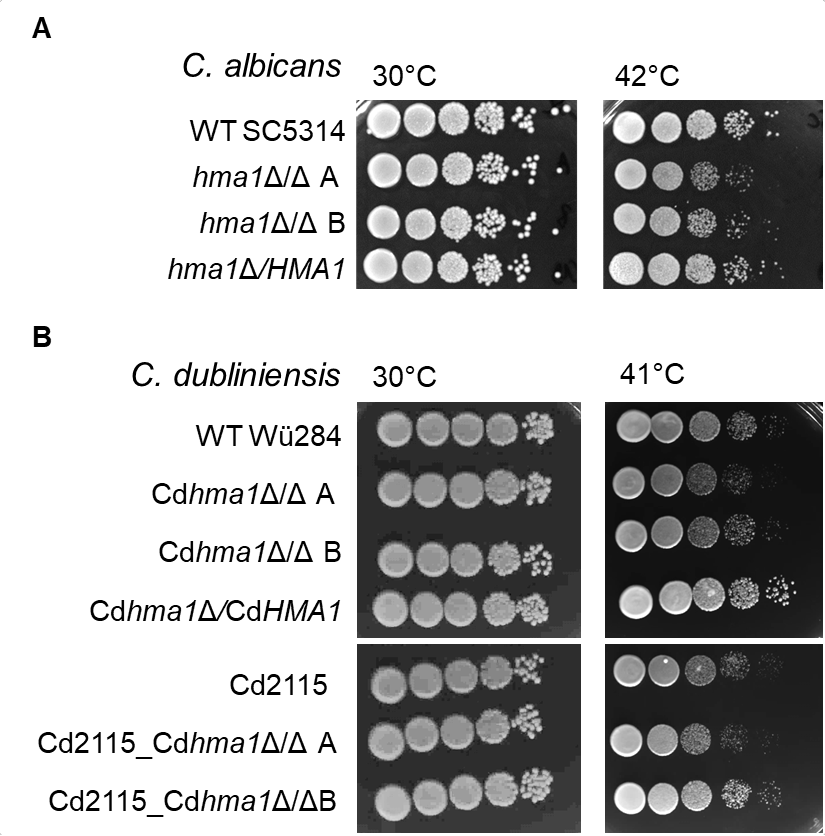


**Figure S5** Serial drop dilution test at elevated temperatures

10^7^ to 10^2^ *Candida* cells were dropped onto YPD-agar plates and incubated for 2 days at standard and elevated temperatures. Heat stress resistance was assayed for *C. albicans* strains (A) at 42°C and for *C. dubliniensis* strains (B) at 41°C.


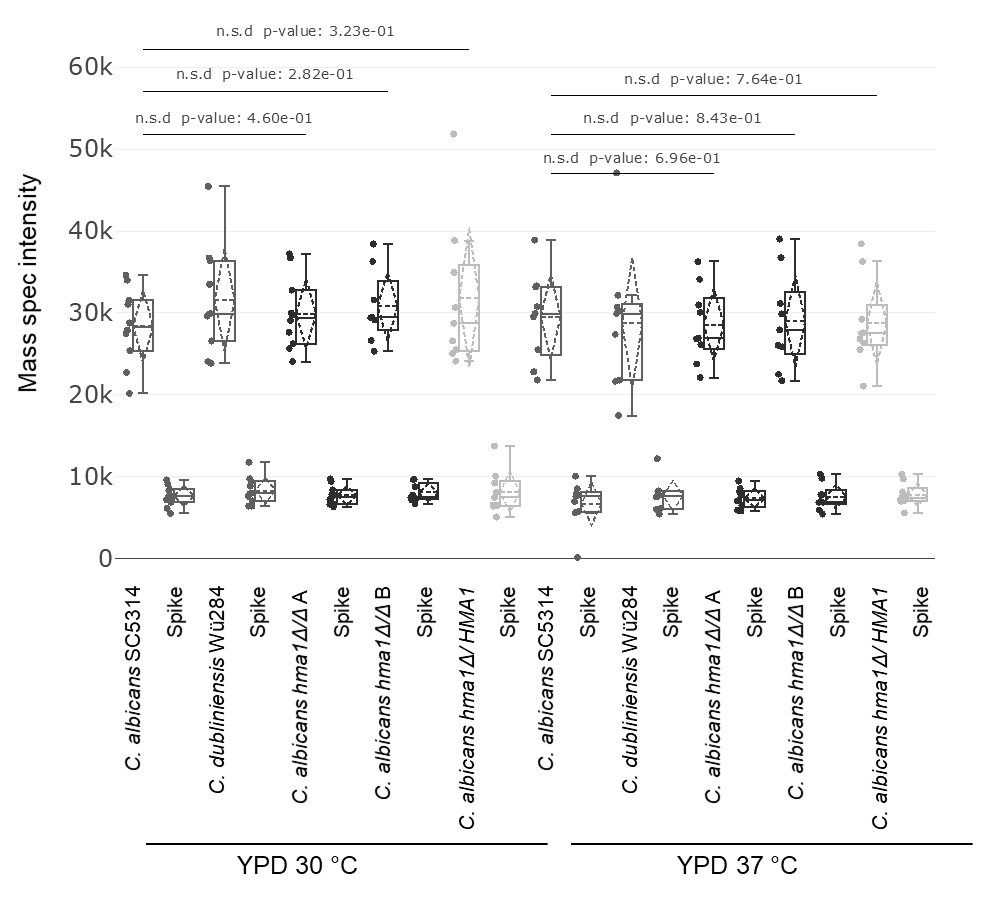
**Figure S6** Adenosine levels in the corresponding tRNA samples. The mass spectrometry intensities of total adenosine levels were constant over all tRNA samples and no statistical differences were determined. (N = three biological replicates)


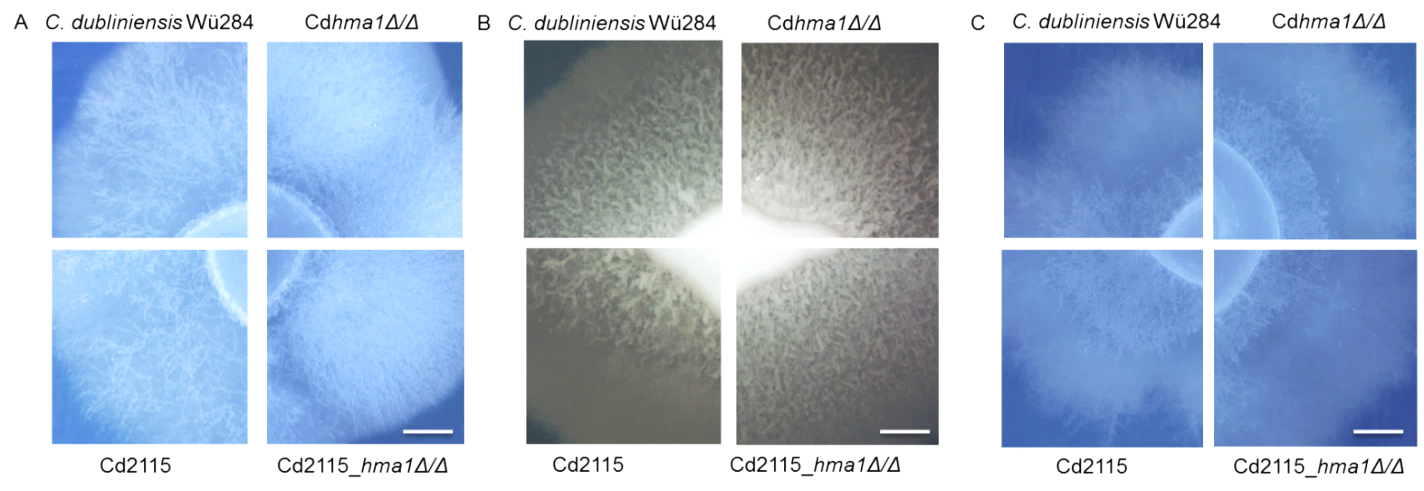


**Figure S7** Filamentation of *C. dubliniensis* strains on solid agar.

*C. dubliniensis* cells from a YPD overnight culture were washed and 10^5^ cells each strain were spotted on (A) SLAD agar, (B) Spider agar, and (C) water agar. Plates were incubated at 37 °C for 14 days. The scale bar represents 1 cm.


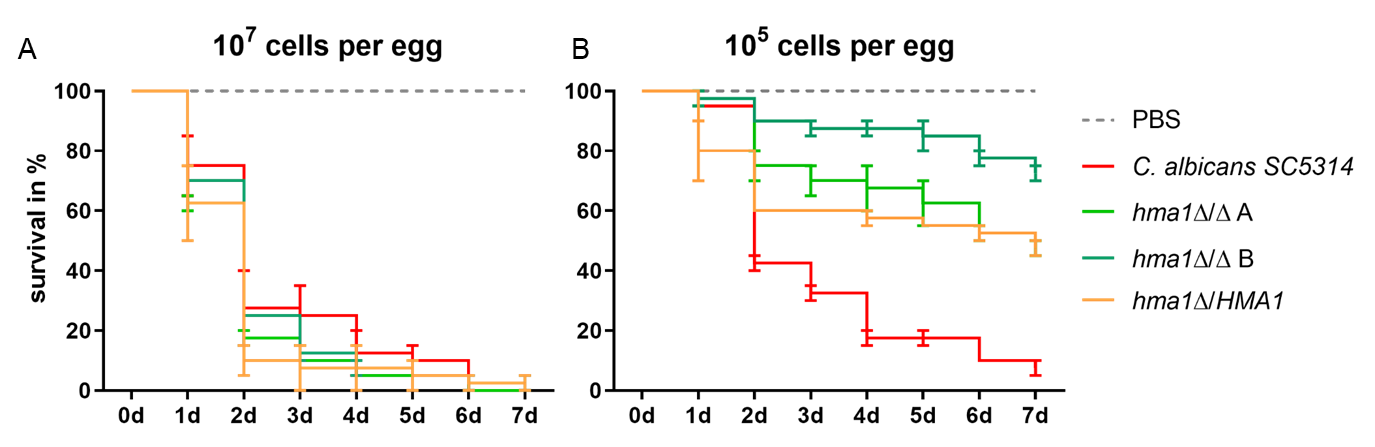


**Figure S8** Virulence of the *C. albicans* wild type strain SC5314, both *hma1*Δ/Δ mutants, and the *hma1*Δ*/HMA1* revertant in infected chicken embryos.

Survival after infection is depicted as Kaplan-Meyer plots. There were 20 chicken embryos per group per experiment, and the combined results of two independent experiments are shown. (A) A high infection dose (10^7^/egg) resulted in high mortality by all *C. albicans* strains. (B) An intermediate infection dose (10^5^/egg) decreased mortality and strain-specific virulence was observed. This graph is identical to figure 7 of the main manuscript and shown here for comparison.
